# P-719. Artificial Intelligence - Driven Syndromic Algorithm for Point-of-Care STI Management: A Randomized Controlled Trial Across Healthcare Tiers

**DOI:** 10.1093/ofid/ofaf695.931

**Published:** 2026-01-11

**Authors:** Debdeep Mitra, Barnali Mitra

**Affiliations:** Command Hospital Air Force Bangalore, Bangalore, Karnataka, India; Command Hospital Western command Chandimandir, Chandigarh, Chandigarh, India

## Abstract

**Background:**

This novel study introduces an AI-integrated syndromic management system for sexually transmitted infections in resource-constrained settings. Our hybrid approach combines traditional WHO syndromic flowcharts with machine learning algorithms trained on region-specific clinical images and patient data. The objective of this study was to evaluate an artificial intelligence system augmenting WHO syndromic protocols for sexually transmitted infection (STI) management in low-resource environments.

**Methods:**

Cluster-randomized controlled trial across 27 Indian facilities (9 urban/9 semi-urban/9 rural) from 2023-2025. The novel AI system combined deep learning image analysis of genital lesions (98,450 curated images) with predictive analytics of symptom patterns (63,120 historical cases). Healthcare workers (n=412) were randomized to AI-assisted (n=14 facilities) or conventional syndromic management (n=13). Primary outcomes: syndrome-specific diagnostic accuracy against NAAT/culture references, antimicrobial appropriateness.

**Results:**

Among 11,902 enrolled patients, AI integration demonstrated:

- 19.3% absolute accuracy improvement for vaginal discharge syndrome versus controls (82.1% vs 62.8%, p< 0.001)
- 39.4% higher mixed-infection detection (71.2% vs 31.8%, p=0.002)
- 14.7% reduction in unnecessary antibiotics (RR 0.73, 95%CI 0.68-0.79)
- 28.5% faster diagnostic decisions in rural clinics (8.2 vs 11.5 minutes, p=0.03)

Image recognition achieved 94.2% sensitivity for herpetic ulcers and 89.7% specificity for syphilitic chancres. The system reduced overtreatment in urban settings by 22.1% while increasing appropriate therapy in rural areas by 31.4%. Cost per quality-adjusted life year (QALY) gained was $14.70, with 92.6% provider adoption sustained at 18 months.

**Conclusion:**

This first AI-syndemic hybrid model significantly outperforms conventional approaches in real-world Indian settings, particularly enhancing vaginal discharge management and antimicrobial stewardship. Its tier-adaptable design provides a scalable solution for Low- and Middle-Income Countries balancing diagnostic accuracy with resource constraints, addressing a critical gap in global STI control strategies.

**Disclosures:**

All Authors: No reported disclosures

